# Impact of the COVID-19 Pandemic on HIV Test Uptake Among People Who Inject Drugs in the Context of an HIV Outbreak

**DOI:** 10.1007/s10461-024-04311-4

**Published:** 2024-04-22

**Authors:** Kirsten M. A. Trayner, Alan Yeung, Norah E. Palmateer, Andrew McAuley, Max Wilkinson, Julie Craik, Rebecca Metcalfe, Erica Peters, Samantha J. Shepherd, Rory N. Gunson, Daniel Carter, Laura Sills, Sharon J. Hutchinson

**Affiliations:** 1 School of Health and Life Sciences, Glasgow Caledonian University, Glasgow, UK; 2 Public Health Scotland, Glasgow, UK; 3 Public Health Protection Unit, NHS Greater Glasgow and Clyde, Glasgow, UK; 4 West of Scotland Specialist Virology Centre, Glasgow, UK; 5 Brownlee Centre for Infectious Diseases, NHS Greater Glasgow and Clyde, Glasgow, UK; 6 NHS Greater Glasgow and Clyde Addiction Services, Glasgow, UK

**Keywords:** HIV testing, People who inject drugs, Data linkage, HIV outbreak, Public health

## Abstract

Glasgow, Scotland’s largest city, has been experiencing an HIV outbreak among people who inject drugs (PWID) since 2015. A key focus of the public health response has been to increase HIV testing among those at risk of infection. Our aim was to assess the impact of COVID-19 on HIV testing among PWID in Glasgow. HIV test uptake in the last 12 months was quantified among: (1) PWID recruited in six Needle Exchange Surveillance Initiative (NESI) surveys (n = 6110); linked laboratory data for (2) people prescribed opioid agonist therapy (OAT) (n = 14,527) and (3) people hospitalised for an injecting-related hospital admission (IRHA) (n = 12,621) across four time periods: pre-outbreak (2010–2014); early-outbreak (2015–2016); ongoing-outbreak (2017–2019); and COVID-19 (2020–June 21). From the pre to ongoing period, HIV testing increased: the highest among people recruited in NESI (from 28% to 56%) and on OAT (from 17% to 54%) while the lowest was among people with an IRHA (from 15% to 42%). From the ongoing to the COVID-19 period, HIV testing decreased markedly among people prescribed OAT, from 54% to 37% (aOR 0.50, 95% CI 0.48–0.53), but increased marginally among people with an IRHA from 42% to 47% (aOR 1.19, 95% CI 1.08–1.31). In conclusion, progress in increasing testing in response to the HIV outbreak has been eroded by COVID-19. Adoption of a linked data approach could be warranted in other settings to inform efforts to eliminate HIV transmission.

## Introduction

The prevalence of HIV among people who inject drugs (PWID) is estimated to be 15.2%, with transmission primarily occurring through the sharing of injecting equipment [1]. The effective prevention of HIV among PWID requires high coverage of HIV prevention services—opioid agonist therapy (OAT), needle and syringe programmes (NSP), and HIV testing followed by access to anti-retroviral therapies (ART). Globally, the coverage of these services for PWID is sub-optimal [2]. In the UK, a lower prevalence of HIV infection (< 2%) has been attributed to the higher coverage of HIV prevention services than reported in other settings internationally [2–4]. Major strides have been made in the prevention and control of HIV both in the UK and globally, resulting in the World Health Organization (WHO) setting targets of eliminating HIV transmission and ending AIDS by 2030 [5, 6]. However, continued transmission among PWID is a barrier to achieving these targets [1]. Effective HIV testing strategies, to reduce undiagnosed infection, are critical to HIV goals [7], but the recent COVID-19 pandemic has presented significant challenges [8, 9].

**Fig. 1:**
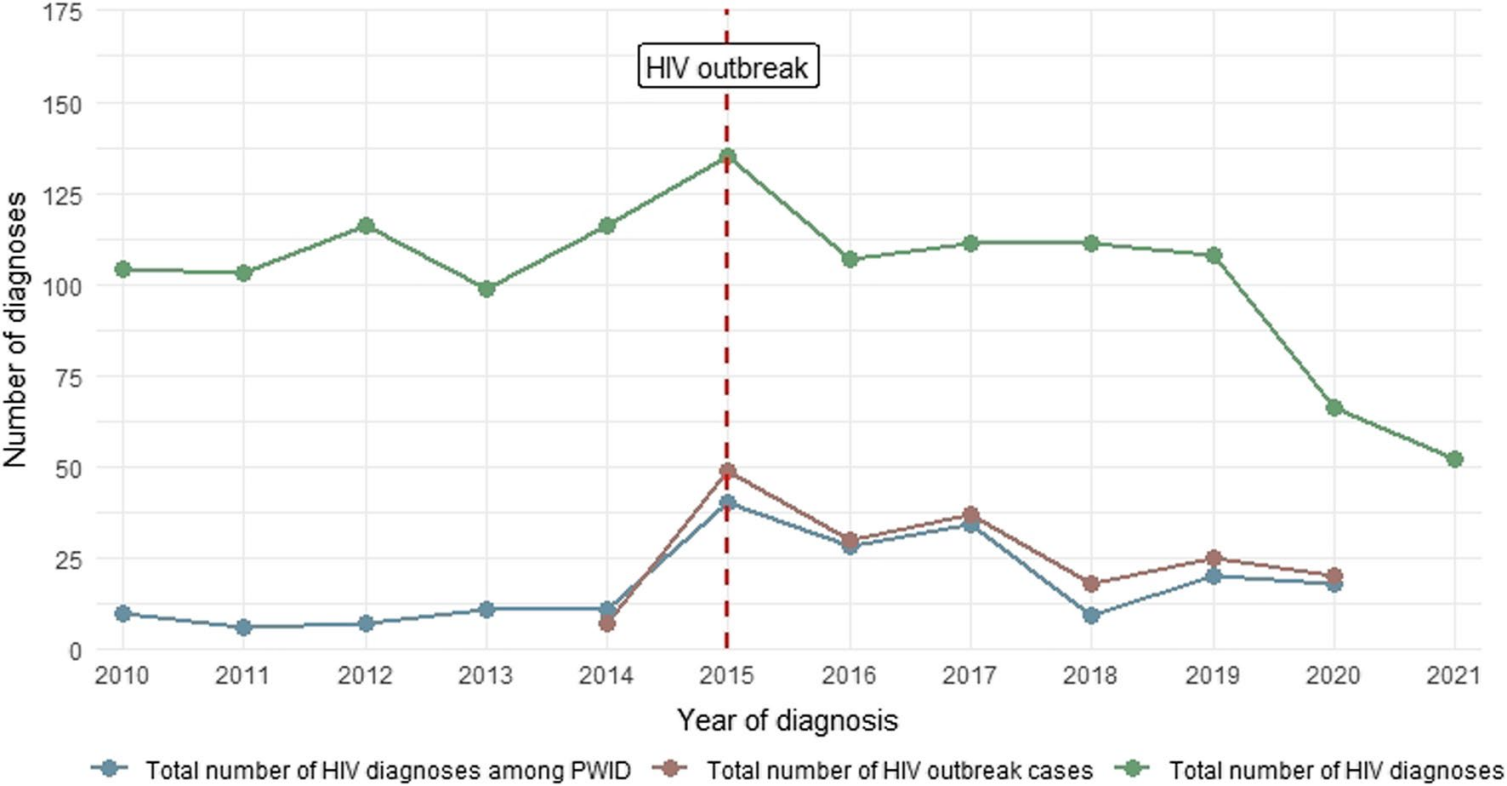
HIV diagnoses in Glasgow, 2010–2021. 2021 data has been removed for deductive disclosure, numbers of cases < 5

The emergence of an HIV outbreak since 2015 in Glasgow, Scotland, which had experienced a low prevalence of HIV among PWID since the 1980’s, underlines the importance of regular surveillance to rapidly identify clusters of undiagnosed infection [10, 11]. HIV outbreaks among PWID have emerged in other settings internationally [12], and limited availability of HIV prevention services (including HIV testing) have been cited as a contributing factor [12–14]. In Glasgow, low HIV testing rates among PWID were regarded as a key factor in the delayed detection and persistence of the outbreak [15]. Glasgow’s public health response—involving the introduction of opt-out blood-borne virus (BBV) testing in prisons and HIV testing on dried blood spot samples from drug services—yielded a doubling in testing coverage among PWID in Glasgow city centre, the epicentre of the outbreak [15]. The emergence of the COVID-19 pandemic has however severely impacted the delivery of HIV testing in Glasgow, and other settings that have experienced HIV outbreaks [16, 17]. There is evidence that the overall number of HIV tests have recovered somewhat, but that gains made pre-pandemic have been eroded [16]. Survey data from elsewhere in the UK also showed decreased HIV testing since the emergence of COVID-19 [18, 19]. Thus, reduced overall testing numbers have resulted in the lower than expected HIV diagnoses in many regions, including Glasgow Scotland [20–23] (Fig. 1).

Reduced contact with PWID has presented not only barriers for delivering HIV testing, but also for surveillance. HIV test uptake (i.e. the proportion of the population who have received a test) among PWID is typically measured using bio-behavioural surveys [24–27]. COVID-19, and related changes in service delivery, have made these traditional methods of data collection more challenging. In Scotland, during the pandemic, all face-to-face data collection was suspended, including the national bio-behavioural survey of PWID known as the Needle Exchange Surveillance Initiative (NESI). Therefore, it was important to consider alternative approaches to measure HIV test uptake. The aim of this study was to explore methods of estimating HIV test uptake among PWID utilising data linkage of routine administrative data. Specific objectives were to: (1) assess the impact of the COVID-19 pandemic on HIV test uptake; and (2) quantify and compare different methods of measuring HIV test uptake among PWID in Glasgow.

## Methods

### Study Design and Data Sources

We assessed HIV test uptake for individuals captured within the following three national datasets held at Public Health Scotland (PHS), which formed our injecting-related cohorts: (1) NESI; (2) OAT prescriptions and (3) injecting-related hospital admissions (IRHA) (summarised in Table 1). We considered data for NHS Greater Glasgow and Clyde (NHS GGC), which represents the largest administrative health area in Scotland and the location of the HIV outbreak.

**Table 1:** Summary of each injecting-related cohort, and outcomes/exposures used to assess HIV test uptake in Glasgow

Injecting-related cohort	Description	Outcomes and exposures
(1) Needle exchange surveillance initiative (NESI) cohort	Study design	Primary outcome
	Repeated cross-sectional bio-behavioural survey	Self-report of HIV test in the last year (yes/no)
	Population	Primary exposure
	Ever or injected drugs in the last 6 months	Time period (pre-outbreak, 2010–2014/early outbreak, 2015–2016/ongoing outbreak, 2017–2019)
	Setting	Secondary exposures
	Harm reduction sites (including drug treatment and needle exchange)	Local authority region^a^ (Glasgow city/rest of Glasgow)
	Data source	Age (< 35/35–45/46 +)
	Needle Exchange Surveillance Initiative (NESI)	Sex (male/female)
	Time period	Self-report of prescribed methadone (not prescribed/in the last 6 months/in the past but not the last 6 months)
	2010–2019	
(2) OAT cohort	Study design	Primary outcome
	Retrospective cohort study constructed using data linkage	HIV test in the last year^c^ (yes/no)
	Population	Primary exposure
	People prescribed OAT^b^	Time period (pre-outbreak, 2010–2014/early outbreak, 2015–2016/ongoing outbreak, 2017–2019/COVID-19, 2020-June 2021)
	Setting	Secondary exposures
	Drug treatment services	Local authority region (Glasgow city/rest of Glasgow)
	Data sources	Age (< 35/35–45/46 +)
	Laboratory HIV test data linked to the Prescribing Information System record of all individuals who received OAT	Sex (male/female)
	Time period	Injecting-related hospital admission in the last 2 years (yes/no)
	2010–June 2021	
(3) Injecting-related hospital admission (IRHA) cohort	Method	Primary outcome
	Retrospective cohort study constructed using data linkage	HIV test in the last year^c^ (yes/no)
	Population	Primary exposure:
	People hospitalised for an injecting-related hospital admission^d^	Time period (pre-outbreak, 2010–2014/early outbreak, 2015–2016/ongoing outbreak, 2017–2019/COVID-19, 2020-June 2021)
	Setting	Secondary exposures:
	Secondary care	Local authority region (Glasgow city/rest of Glasgow)
	Data source	Age (< 35/35–45/46 +)
	Laboratory HIV test data linked to the Scottish Mortality Record 01 record of all individuals who have been hospitalised for an injecting-related hospital admission^d^	Sex (male/female)
	Time period	Prescribed OAT (not prescribed/in the last 6 months/in the past but not the last 6 months)
	2010–June 2021	

*NESI* Needle Exchange Surveillance Initiative, *OAT* Opioid agonist therapy, *IRHA* Injecting-related hospital admission

^a^Based on region of recruitment in the NESI study

^b^Prescribed methadone, buprenorphine and buprenorphine/naloxone

^c^Laboratory record of an HIV test in the last year, relative to last OAT prescription date or to last IRHA date for each respective calendar period

^d^Injecting-related hospital defined used ICD-10 codes included in Appendix, Table 4

The NESI cohort included PWID recruited as part of a repeated cross-sectional bio-behavioural survey conducted biennially in Scotland, involving six sweeps during 2010–2019; data relating to the COVID-19 period were not available. Participants who had ever injected were recruited from services providing injecting equipment and other harm reduction services across Scotland (thus 70–80% of participants had injected in the last 6 months). Full NESI methods are described elsewhere [15, 25].

The OAT and IRHA cohorts were constructed using a retrospective cohort study design and data linkage. The Prescribing Information System (PIS) dataset was used to form the OAT cohort, which contains a record of all drugs which are paid for, prescribed and dispensed in the community in Scotland [28]. Data were extracted relating to the prescription of OAT for opioid dependence (i.e. methadone, buprenorphine and buprenorphine/naloxone) [29]. The Scottish Mortality Record 01 (SMR01) formed the IRHA cohort. SMR01 is a national database of all individuals who have been admitted to hospital and received secondary care in Scotland. Individuals who had been hospitalised for an IRHA from 2010 to June 2021 were identified using International Classification of Disease codes, relating to drugs known to be injected in Scotland and injecting-related infections most described in the literature [30, 31] (Appendix, Table 4).

All individuals in Scotland who have accessed healthcare are allocated a unique identifier, a Community Health Index (CHI) number [32]. Both the PIS and SMR01 data were linked to the outcome dataset, which was all laboratory HIV tests conducted in NHS GGC during the study period using CHI. HIV test data was obtained from the NHS West of Scotland Specialist Virology Centre who provide specialist HIV testing in NHS GGC. This included information on all HIV antigen/antibody initial screens for new diagnoses, confirmation testing (HIV-1/HIV-2 antibody assays, HIV avidity testing) and PCR tests to monitor individuals receiving ART. Only tests which related to screens for new diagnoses were retained (i.e. confirmation and treatment monitoring tests were removed). We received Caldicott Guardian approval from NHS GGC to transfer the laboratory HIV test data to PHS, and the linkage and analysis of data held at PHS received approval from the NHS Scotland Public Benefit and Privacy Panel for Health and Social Care (PBPP 2021-0203).

### Outcomes and Exposures

The primary outcome measure was uptake of an HIV test in the last year. Within the NESI cohort, this was based on self-report of an HIV test, and individuals who self-reported HIV infection but did not self-report an HIV test in the last year (i.e. ineligible for repeat testing) were removed. Within the OAT and IRHA cohorts, being tested for HIV in the last year was calculated relative to their last OAT prescription date or last IRHA date for each respective time period. Relating to the OAT cohort, for those who had an OAT prescription after their date of death (OAT prescriptions often cover 14–28 days), their most recent prescription date prior to their date of death was selected. People who had been diagnosed with HIV more than a year prior to their last OAT prescription date or last IRHA date for each time period (i.e. ineligible for repeat testing) were removed from each OAT/IRHA cohort (Table 1).

The primary exposure was time period; testing was assessed across four periods: pre-outbreak (2010–2014); early outbreak (2015–2016), ongoing outbreak (2017–2019); COVID-19 (2020–June2021). Key secondary exposures included local authority region within NHS GGC (Glasgow city/rest of Glasgow), age (< 35/35–45/46 +), IRHA in the last 2 years (yes/no), and OAT/methadone prescribing (not prescribed/prescribed in the last 6 months/prescribed but not in the last 6 months). For the OAT and IRHA cohorts, their first recorded local authority record was selected. Relating to NESI, local authority was based on region of recruitment. Secondary exposures varied for each cohort, depending on data availability (Table 1).

### Statistical Analysis

For each cohort, HIV test uptake in the last year was first quantified by time period and local authority. Multi-variate logistic regression was used to assess changes in HIV testing across time periods in each injecting-related cohort and local authority region. Previous research has shown the public health response to the outbreak increased HIV testing in Glasgow [15], therefore the ongoing outbreak period (2017–2019) was used as the reference category to capture the impact of the pandemic on testing. Time period was a time varying co-variate (i.e. individuals included in each cohort could be included in multiple time periods). To account for the presence of individuals across multiple time periods for each cohort, a multi-level framework was applied to logistic regression models [15, 33, 34]. Analysis was undertaken using Stata 13.

### Post-hoc Analysis

We conducted a post-hoc analysis to investigate why test uptake increased in the IRHA cohort, but decreased in the OAT cohort, in the COVID-19 period (2020–June2021) relative to the ongoing outbreak period (2017–2019). Within the OAT cohort, we included an interaction between time period and being hospitalised for an IRHA in the last 2 years.

## Results

### Cohort Characteristics

A total of 6100 participants were included in the NESI cohort, 14,527 and 12,621 people were included in the OAT and IRHA cohort, respectively. The majority of the NESI and OAT cohort were included pre-outbreak (2010–2014) (NESI cohort: 54%, n = 3302; OAT cohort: 84%, n = 11,908), whereas the majority of the IRHA cohort were included in the ongoing outbreak period (2017–2019) (42%, n = 5297). Furthermore, the majority were also included in the Glasgow city local authority region (NESI cohort: 75%, n = 4586; OAT cohort: 73%, n = 10,466; IRHA cohort: 65%, n = 8178) and male (NESI cohort: 73%, n = 4465; OAT cohort: 69%, n = 9826; IRHA cohort: 71%, n = 8942). In relation to age, most NESI participants were aged 35–45 in each time period. An increasing age for each time period was observed among those included in the OAT and IRHA cohort. Among the OAT cohort, 22% (n = 3187) had an IRHA in the last 2 years and 40% (n = 5064) of the IRHA had received OAT in the last 6 months (relative to their last prescription or admission date for the whole study period, respectively) (Table 2).

**Table 2:** Participants characteristics in each injecting-related cohort, 2010–June 2021

Co-variates	Injecting-related cohort		
	Needle exchange surveillance initiative cohort (NESI)^a^ (% of N)	Opiate agonist therapy (OAT) cohort (% of N)	Injecting-related hospital admission (IRHA) cohort (% of N)
Total, N	6,110	14,257	12,621
Time period^b^			
Pre-outbreak (2010–2014)	3,302 (54%)	11,908 (84%)	4,826 (38%)
Early outbreak (2015–2016)	940 (15%)	9,519 (67%)	3,347 (27%)
Ongoing outbreak (2017–2019)	1,868 (31%)	9,412 (66%)	5,297 (42%)
COVID-19 (2020–June 2021)	–	7,599 (53%)	3,008 (24%)
Local authority area			
Glasgow city	4,586 (75%)	10,466 (73%)	8,178 (65%)
Rest of Glasgow	1,524 (25%)	3,791 (27%)	4,443 (35%)
Not recorded/unknown	0	0	0
Gender			
Male	4,465 (73%)	9,826 (69%)	8,942 (71%)
Female	1,618 (26%)	4,431 (31%)	3,679 (29%)
Not recorded/unknown	27 (1%)	0	0
Age group, pre-outbreak (2010–2014)			
< 35	1,385 (42%)	3,170 (27%)	2,079 (43%)
35–45	1,613 (49%)	5,661 (47%)	1,705 (35%)
46 +	301 (9%)	3,077 (26%)	1,042 (22%)
Not recorded/unknown	3 (< 1%)	0	0
Age group, early outbreak (2015–2016)			
< 35	273 (29%)	1,667 (18%)	1,190 (36%)
35–45	479 (51%)	4,588 (48%)	1,179 (35%)
46 +	188 (20%)	3,264 (34%)	978 (29%)
Not recorded/unknown	0	0	0
Age group, ongoing outbreak (2017–2019)			
< 35	297 (16%)	1,146 (12%)	1,784 (34%)
35–45	1,024 (55%)	3,939 (42%)	1,703 (32%)
46 +	544 (29%)	4,327 (46%)	1,810 (34%)
Not recorded/unknown	3 (< 1%)	0	0
Age group, COVID-19 (2020–2021)			
< 35	–	755 (10%)	911 (30%)
35–45	–	2,893 (38%)	950 (32%)
46 +	–	3,951 (52%)	1,147 (38%)
Not recorded/unknown	–	0	0
Prescribed OAT^c,d^			
Not recorded/unknown			
Not prescribed OAT	426 (7%)	–	6,552 (52%)
In the last 6 months	5,039 (82%)	14,527 (100%)^e^	5,064 (40%)
In the past but not the last 6 months	607 (10%)	–	1,005 (8%)
Not recorded/unknown	38 (1%)	–	0
Injecting-related hospital admission in the last 2 years^d^			
Yes	–	3,187 (22%)	12,621 (100%)^e^
No	–	11,070 (78%)^e^	–
Not recorded/unknown	–	0	–

*NESI* Needle Exchange Surveillance Initiative, *OAT* Opioid agonist therapy, *IRHA* Injecting-related hospital admission

^a^NESI cohort is described by participations in the NESI survey

^b^Relates to recruitment period for NESI cohort; Time-varying co-variate for OAT and IRHA, people can be included in multiple time periods

^c^NESI cohort: prescribed methadone

^d^OAT cohort, relative to last OAT prescription date; IRHA cohort: relative to date of last hospital admission

^e^Definition of cohort (OAT prescription or IRHA) translates to 100% for these categories

### HIV Test Uptake by Time Period and Local Authority Region

**Fig. 2:**
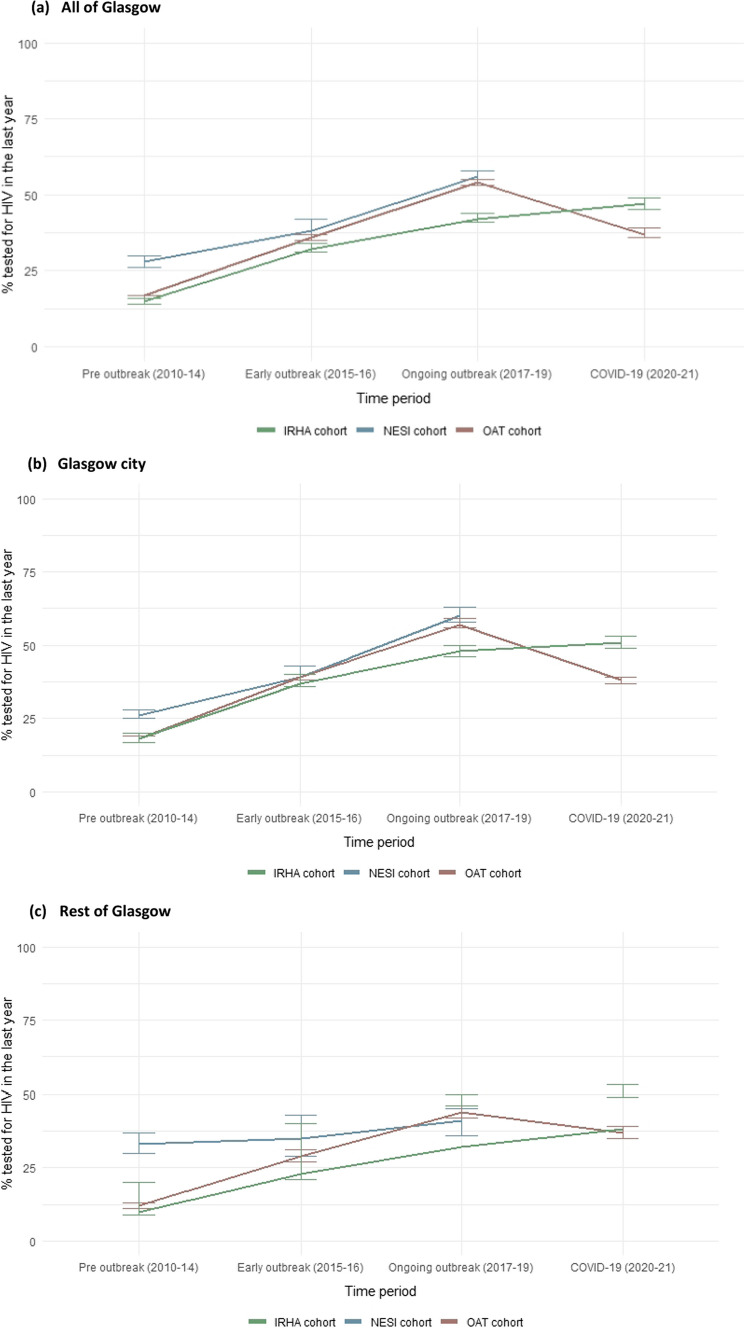
HIV test uptake in the last year in each injecting-related cohort, 2010-June 2021. **a** All of Glasgow, **b** Glasgow city, **c** Rest of Glasgow

From the pre-outbreak period (2010–2014) to the ongoing outbreak period (2017–2019), the trend in the uptake of HIV testing consistently increased in each injecting-related cohort. However, there were differences in uptake, with the highest proportions observed in the NESI cohort (28% in pre-outbreak period to 56% in the ongoing outbreak period) and the lowest among the IRHA cohort (15% to 42%, respectively) (Fig. 2a).

Within the OAT cohort, test uptake decreased to 37% during the COVID-19 period (2020–June 2021). Conversely, test uptake increased in the COVID-19 (2020–June 2021) period in the IRHA cohort to 47% (Fig. 2a). Similar trends for each cohort were observed among those recruited in Glasgow city and rest of Glasgow, however, the proportion tested in each period was highest in Glasgow city and lowest in the rest of Glasgow (Fig. 2b, c).

### HIV Test Uptake by Time Period: Multi-variate Analysis

Among those included in the NESI cohort, when compared to the reference category of the ongoing outbreak period (2017–2019), there were reduced odds of being tested in the early outbreak period (2015–2016) (aOR 0.47, 95% CI 0.40–0.55, p < 0.001) and the pre-outbreak period (2010–14) (aOR 0.28, 95% CI 0.25–0.32, p < 0.001) (Table 3). Similar findings were observed among those recruited in Glasgow city (Appendix, Table 6); however, a significant difference was not observed in the early outbreak period (2015–2018) among those recruited in the rest of Glasgow (Appendix, Table 7).

**Table 3:** Univariate and multi-variate models assessing uptake of an HIV test in each injecting-related cohort in all of Glasgow, 2010–June 2021

Time period	N	HIV test in the last year (% of N)	Univariate OR^a^ (95% CI)	P-value	Multi-variate aOR^a^ (95% CI)	P-value
Needle exchange surveillance initiative cohort^b^						
Pre HIV outbreak (2010–2014)	3,139	889 (28%)	0.32 (0.28-0.36)	< 0.001	0.28 (0.25 to 0.32)	< 0.001
Early HIV outbreak (2015–2016)	886	341 (38%)	0.49 (0.42–0.59)	< 0.001	0.47 (0.40–0.55)	< 0.001
Ongoing HIV outbreak (2017–2018)	1,802	1,002 (56%)	1		1	
COVID-19 (2020–2021)	–	–	–	–	–	–
Opiate agonist therapy cohort^c^						
Pre HIV outbreak (2010–2014)	11,908	1,973 (17%)	0.17 (0.16–0.18)	< 0.001	0.16 (0.15–0.17)	< 0.001
Early HIV outbreak (2015–2016)	9,519	3,430 (36%)	0.48 (0.46–0.51)	< 0.001	0.47 (0.44–0.49)	< 0.001
Ongoing HIV outbreak (2017–2018)	9,412	5,053 (54%)	1		1	
COVID-19 (2020–2021)	7,599	2,847 (37%)	0.52 (0.49–0.55)	< 0.001	0.50 (0.48–0.53)	< 0.001
Injecting-related hospital admission cohort^d^						
Pre HIV outbreak (2010–2014)	4,826	737 (15%)	0.24 (0.22–0.27)	< 0.001	0.19 (0.17–0.21)	< 0.001
Early HIV outbreak (2015–2016)	3,347	1,082 (32%)	0.64 (0.59–0.71)	< 0.001	0.54 (0.49–0.60)	< 0.001
Ongoing HIV outbreak (2017–2019)	5,297	2,248 (42%)	1		1	
COVID-19 (2020–2021)	3,008	1,411 (47%)	1.20 (1.10–1.30)	< 0.001	1.19 (1.08–1.31)	0.001

*OR* odds ratio, *aOR* adjusted odds ratio

^a^Multi-level framework applied to adjust for duplicates

^b^Adjusted for: calendar period (excluding COVID-19 period), local authority, age, sex, prescribed methadone

^c^Adjusted for: calendar period, local authority, age, sex, recent drug-related hospital admission (last 2 years)

^d^Adjusted for: calendar period, local authority, age, sex, prescribed OAT

**Fig. 3:**
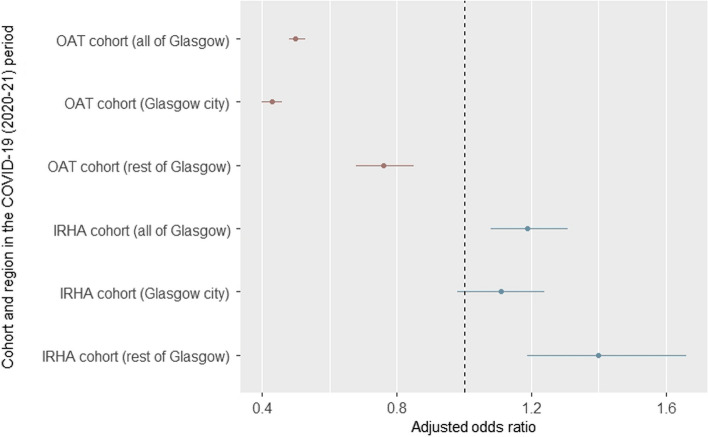
Impact of the COVID-19 pandemic* on HIV test uptake among people prescribed OAT and people hospitalised for an injecting-related hospital admission (IRHA) in all of Glasgow, and stratified by Glasgow city and rest of Glasgow. *Relative to HIV test uptake in the ongoing outbreak period (2017–2019). *OAT* opioid agonist therapy, *IRHA* injecting-related hospital admission

Among those included in the OAT and IRHA cohort, we also observed a reduced odds of being tested pre-outbreak (2010–2014) (OAT cohort: aOR 0.16, 95% CI 0.15–0.17, p < 0.001; IRHA cohort: aOR 0.19; 95% CI 0.17–0.21;p < 0.001) and early outbreak (2015–2016) (OAT cohort: aOR 0.47, 95% CI 0.44–0.49, p < 0.001; IRHA cohort: aOR 0.54, 95% CI 0.49–0.60, p < 0.001), relative to ongoing outbreak period (2017–2019). Reduced odds of being tested was observed in the COVID-19 period (2020–June 2021) for those in the OAT cohort (aOR 0.50, 95% CI 0.48–0.53, p < 0.001) and increased odds of being tested were observed in the IRHA cohort (aOR 1.19, 95% CI 1.08–1.31, p < 0.001), relative to the ongoing outbreak period (2017–2019) (Table 3, Fig. 3). Similar findings were observed among both the OAT cohort and IRHA cohort in the rest of Glasgow (Fig. 3, Appendix, Tables 10, 13). However, we did not observe a significant difference in test uptake in the IRHA cohort in the COVID-19 period (2020-June 2021) in Glasgow city (aOR 1.11, 95% CI 0.98–1.24, p = 0.080) (Fig. 3, Appendix, Table 12). Full models for each injecting-related cohort, stratified by local authority region, can be found in Appendix, Tables 5, 6, 7, 8, 9, 10, 11, 12, 13.

### Post-hoc Analyses

To explore further the different trends in HIV test uptake in the COVID-19 period between OAT and IRHA cohorts, we considered an interaction between time period and being hospitalised for an IRHA in the last 2 years within the OAT cohort. We found an increased odds of being tested in the COVID-19 period (2020-June 2021) for those who had been hospitalised with a recent IRHA (aOR 3.15, 95% CI 2.81 to 3.51, < 0.001) and a reduced odds for those who had not been hospitalised for a recent IRHA (aOR 0.47, 95% CI 0.44 to 0.51, < 0.001), relative to those without a recent admission in the ongoing outbreak period (2017–2019) (Appendix, Table 14).

## Discussion

In the context of an HIV outbreak among PWID, our aim was to explore methods of estimating HIV test coverage, and assess the impact of the COVID-19 pandemic on HIV testing among PWID in Glasgow. Utilising linkage of routine administrative and bio-behavioural survey data, we found that pre-pandemic, HIV test uptake was increasing across all cohorts due to the focus on testing as part of the outbreak response. However, findings from the linked administrative analysis also suggest that the considerable progress in increasing HIV test uptake has been impacted by the pandemic, which could have implications for national policy goals to eliminate HIV transmission and end AIDS by 2030.

We found that COVID-19 negatively impacted HIV test uptake among people prescribed OAT in Glasgow, suggesting that the substantial progress observed pre-pandemic in response to the outbreak has been eroded [15]. The decrease in test uptake among people prescribed OAT is not unexpected due to changes in the delivery of drug treatment, including take-home OAT doses and long acting injectable OAT, which have reduced contact with people who use drugs and therefore opportunities to deliver testing [16]. The introduction of take-home OAT and long acting injectable OAT have also been reported in other regions globally [35], with studies from settings including Spain, Australia and USA reporting no unintended consequences on drug-related outcomes such as mortality [36–39]. However, the resulting reduced contact between PWID and service providers has unintended consequences for the delivery of HIV prevention services. Reduced testing and treatment coverage during the pandemic have not only been reported among PWID, but other populations at risk of HIV, such as men who have sex with men [20, 21, 40–43].

Reduced testing may have resulted in lower than expected HIV diagnoses in many regions [20–23]. Relating to diagnoses associated with HIV outbreak, over 20 HIV cases were diagnosed in Glasgow in 2020, mainly pre-lockdown (March 2020), but that number has significantly reduced in 2021 to 22 diagnoses (to below five at the time of writing) (Fig. 1), which could be related to reduced testing levels. Lower than expected HIV diagnoses, combined with reduced routine HIV testing among people prescribed OAT could suggest clusters of undiagnosed infection. The impact of COVID-19 on transmission related to the HIV outbreak in Glasgow and other settings remains unknown [17]. A study from British Columbia, Canada found increased HIV transmission clusters associated with reduced access to health services, particularly among PWID, where clusters showed rapid growth and compared to other at risk groups [42]. The latest data on the epidemiology of the HIV outbreak relates to 2019 (the last NESI sweep), which suggested that HIV transmission had been contained in Glasgow city centre, but was increasing in areas surrounding Glasgow. The lower test uptake in the rest of Glasgow (35%), relative to Glasgow city (37%), is concerning in this context and suggests that previous trends—i.e. a higher uptake in the city centre in contrast to the rest of Glasgow—are continuing [15]. Further research is required to assess whether differences in the coverage of services, including testing, has contributed to the spread of HIV outside of Glasgow city. Whilst linked data can provide intelligence on the impact of COVID-19 on testing coverage, enhanced surveillance through NESI is required to provide crucial epidemiological information on the impact of the pandemic on HIV transmission in Scotland.

The increase in HIV testing among people hospitalised for an IRHA highlights a successful COVID-19 mitigation strategy, where people with a history of drug use continued to be tested if admitted to hospital [16]. Hospitals have been a key test setting over the course of the outbreak, and the majority of PWID diagnosed as part of the outbreak were diagnosed in secondary care, and over 70% of diagnosed HIV outbreak cases between 2015 and 2019 had an acute presentation in a hospital setting prior to diagnosis [44]. Although secondary care is an important setting, community-based BBV testing alongside other harm reduction services should ideally pick up the vast majority of cases, with a minority being diagnosed in settings such as secondary care. Community-based testing strategies have been key to improving test uptake in other settings that have experienced HIV outbreaks [12], and thus further intervention is required to improve community test coverage in Glasgow, and across Scotland. Contingency management (i.e. the provision of financial incentives), has shown promise in engaging PWID in HIV prevention and care [45], and has recently been introduced in Glasgow to target testing among high risk PWID. Opt-out testing policies in settings attended by PWID have also been shown to be effective in increasing testing coverage, particularly drug, prison, harm reduction and social services [4, 15, 46, 47]. In Scotland, opt-out testing in drug services by the end of 2024 is a key recommendation that is part of the new medication assisted treatment standards [48]. Other settings have been considered for opt out testing, including GPs and emergency departments, which are important for not only the diagnosis of PWID, but other at risk groups, and have shown promising findings in England [49]. Further research is required to better identify factors associated with undiagnosed HIV infection and late diagnosis of HIV, to inform efficient testing strategies.

Effective HIV testing strategies, including increased avidity testing to track how quickly infections are being diagnosed, are not only instrumental for the control of the HIV outbreak, but for national and international policy objectives to eliminate HIV transmission and end AIDS by 2030 [50]. In addition to impacting HIV testing rates, the pandemic has also affected traditional methods to measure HIV test uptake, including the delivery of bio-behavioural surveys [24–27]. We have shown that data linkage of routine administrative data sources is broadly consistent to bio-behavioural surveys in relation to trends, but differences are observed in relation to uptake. This highlights how different groups of PWID are more or less likely to be tested, and thus a range of testing and surveillance approaches are required. We have explored other methods of measuring test uptake using data linkage, that could also be applied to other populations groups, and infections, such as HCV. Furthermore, these methods could be particularly insightful in settings without bio-behavioural surveillance to monitor progress and identify gaps in testing uptake as we move towards both HCV and HIV transmission elimination.

## Strengths and Limitations

Response bias is a limitation of self-reported survey data. However, we quantified HIV test uptake using different methods and data sources, and observed broadly similar findings between self-reported NESI data and linked administrative data pre-pandemic, reflecting the focussed efforts to scale up testing in drug services as part of the outbreak response. Another limitation of our work is that we could not assess HIV test uptake nationally, where it would have been beneficial to compare testing in Glasgow to other regions in Scotland that have not experienced an outbreak. Individuals who live in Glasgow may also have been tested outside of Glasgow. The data linkage relied on the availability of CHI on administrative data to link records. Records with a CHI number in the hospital admissions data are high (over 95%). However, among those prescribed OAT, approximately 75–80% of methadone and buprenorphine prescriptions included a CHI number from 2015 to 2020. While this includes most people prescribed OAT, it will not cover the entirety of people receiving OAT, including those who have received OAT in non-community settings such as prisons. Similarly, HIV test records missing CHI numbers or identifiable information may also not captured. This includes testing in sexual health services in Scotland which is anonymised, and testing conducted by third sector partners who do not routinely record CHI numbers so test uptake may be slightly underestimated.

## Conclusion

Our findings highlight how progress in increasing testing coverage among PWID in response to the HIV outbreak has been eroded as a result of the pandemic, particularly among people prescribed OAT. The linkage of administrative data on people in contact with drug services provided key intelligence, complimenting data generated from self-reported survey data, to monitor test uptake among PWID. In the context that most countries lack national bio-behavioural surveys of PWID, the adoption of a similar linkage approach is warranted in other international settings to monitor and inform testing efforts to support ambitions to eliminate HIV transmission and end AIDS in this population.

## Appendix

See Tables 4, 5, 6, 7, 8, 9, 10, 11, 12, 13, 14.

**Table 4:** Definition of an injecting-related hospital admission using International Classification of Disease (ICD) 10 codes

ICD-10 code	Description
F11	Mental and behavioural disorders due to: opioids
F13	Mental and behavioural disorders due to: sedatives/hypnotics
F14	Mental and behavioural disorders due to: cocaine
F15	Mental and behavioural disorders due to: other stimulants
F19	Mental and behavioural disorders due to: multiple/other drugs
T40.0	Poisoning by narcotics: Opium
T40.1	Poisoning by narcotics: Heroin
T40.3	Poisoning by narcotics: Methadone
T40.5	Poisoning by narcotics: Cocaine
T40.6	Poisoning by narcotics: Unspecified narcotics
Codes below must also have codes F11, F13-15, F19 in the same CIS	
T40.2	Poisoning by narcotics: other opioids
T42.2	Poisoning by antiepileptic, sedative-hypnotic and antiparkinson drugs: benzodiazapines
I33	Acute and subacute infective endocarditis
L02	Cutaneous abscess, furuncle and carbuncle, unspecified
L03	Cellulitis
L08	Other local infections of skin and subcutaneous tissue
I80	Phlebitis and thrombophlebitis
A40	Septicaemia due to streptococcus
A41	Other sepsis
A49.0, A49.1	Staphylococcal or streptococcal of unspecified site
M86	Osteomyelitis
A35	Other tetanus
M72.6	Necrotizing fasciitis
R22.2–R22.9	Localised swelling, mass and lump on neck, trunk, upper limb, lower limb, multiple sites or unspecified
L97	Ulcer of lower limb, not elsewhere classified
L98.8	Other disorders of skin and subcutaneous tissue, not elsewhere classified: Other specified disorders of skin and subcutaneous tissue
L98.4	Chronic ulcer of the skin, not elsewhere specified
M79.8, M79.9	Other or unspecified soft tissue disorder

**Table 5:** Univariate and multi-variate models assessing HIV testing rates among people recruited as part of the *Needle Exchange Surveillance Initiative* in all of Glasgow, 2010–2019

Co-variates	N^a^	HIV test in the last year (% of N)	Univariate^b^		Multi-variate^b^	
			OR (95% CI)	P value	aOR (95% CI)	P value
Time period						
Pre-outbreak (2010–2014)	3,139	889 (28%)	0.32 (0.28–0.36)	< 0.001	0.28 (0.25–0.32)	< 0.001
Early outbreak (2015–2016)	886	341 (38%)	0.49 (0.42–0.59)	< 0.001	0.47 (0.40–0.55)	< 0.001
Ongoing outbreak (2017–2019)	1,802	1,002 (56%)	1		1	
Local authority area						
Rest of Glasgow	1,438	514 (36%)	1		1	
Glasgow city	4,389	1,718 (39%)	1.16 (1.02–1.31)	0.024	1.17 (1.02–1.34)	0.029
Gender						
Male	4,246	1,608 (38%)	1		1	
Female	1,556	613 (39%)	1.07 (0.94–1.21)	0.308	1.03 (0.91–1.18)	0.609
Age group						
< 35	1,856	687 (37%)	1		1	
35–45	2,972	1,115 (37%)	1.02 (0.90–1.15)	0.731	0.74 (0.65–0.85)	< 0.001
46 +	993	428 (43%)	1.29 (1.09–1.51)	0.002	0.72 (0.59–0.85)	< 0.001
Methadone prescribing						
Not prescribed	419	108 (25%)	1		1	
In the last 6 months	4,803	1,894 (39%)	1.87 (1.49–2.35)	< 0.001	2.34 (1.86–2.95)	< 0.001
In the past but not the last 6 months	578	221 (38%)	1.78 (1.35–2.34)	< 0.001	2.25 (1.70–2.98)	< 0.001

*OR* odds ratio, *aOR* adjusted odds ratio

^a^Missing data has been excluded

^b^Multi-level framework to adjust for across survey duplicates

**Table 6:** Univariate and multi-variate models assessing HIV testing rates among people recruited as part of the *Needle Exchange Surveillance Initiative* in Glasgow city, 2010–2019

Co-variates	N^a^	HIV test in the last year (% of N)	Univariate^b^		Multi-variate^b^	
			OR (95% CI)	P value	aOR (95% CI)	P value
Time period						
Pre-outbreak (2010–2014)	2,304	610 (26%)	0.24 (0.21–0.27)	< 0.001	0.21 (0.17–0.24)	< 0.001
Early outbreak (2015–2016)	700	275 (39%)	0.42 (0.36–0.51)	< 0.001	0.39 (0.33–0.48)	< 0.001
Ongoing outbreak (2017–2019)	1,385	833 (60%)	1		1	
Gender						
Male	3,245	1,266 (39%)	1		1	
Female	1,124	444 (40%)	1.02 (0.88–1.17)	0.781	0.96 (0.82–1.12)	0.618
Age group						
< 35	1,297	482 (37%)	1		1	
35–45	2,251	861 (38%)	1.05 (0.91–1.21)	0.530	0.73 (0.62–0.85)	< 0.001
46 +	835	373 (45%)	1.36 (1.13–1.64)	0.001	0.65 (0.53–0.80)	< 0.001
Methadone prescribing						
Never prescribed	342	89 (26%)	1		1	
In the last 6 months	3,545	1,436 (41%)	1.93 (1.51–2.48)	< 0.001	2.45 (1.89–3.18)	< 0.001
In the past but not the last 6 months	482	184 (38%)	1.76 (1.29–2.37)	< 0.001	2.40 (1.76–3.28)	< 0.001

*OR* odds ratio, *aOR* adjusted odds ratio

^a^Missing data has been excluded

^b^Multi-level framework to adjust for across survey duplicates

**Table 7:** Univariate and multi-variate models assessing HIV testing rates among people recruited as part of the *Needle Exchange Surveillance Initiative* in rest of Glasgow, 2010–2019

Co-variates	N^a^	HIV test in the last year (% of N)	Univariate^b^		Multi-variate^b^	
			OR (95% CI)	P value	aOR (95% CI)	P value
Time period						
Pre-outbreak (2010–2014)	835	279 (33%)	0.74 (0.57–0.94)	0.014	0.67 (0.52–0.87)	0.002
Early outbreak (2015–2016)	186	66 (35%)	0.81 (0.56–1.15)	0.236	0.79 (0.55–1.15)	0.220
Ongoing outbreak (2017–2019)	417	169 (41%)	1		1	
Gender						
Male	1,001	342 (34%)	1		1	
Female	432	169 (39%)	1.24 (0.97–1.57)	0.081	1.23 (0.96–1.57)	0.102
Age group						
< 35	559	205 (37%)	1		1	
35–45	721	254 (35%)	0.94 (0.75–1.18)	0.595	0.85 (0.67–1.09)	0.207
46 +	158	55 (35%)	0.92 (0.64–1.33)	0.664	0.84 (0.56–1.24)	0.371
Methadone prescribing						
Not prescribed	77	19 (25%)	1		1	
In the last 6 months	1,258	458 (36%)	1.75 (1.04–2.95)	0.036	1.93 (1.14–3.28)	0.014
In the past but not the last 6 months	96	37 (38%)	1.91 (0.98–3.73)	0.057	2.11 (1.08–4.15)	0.029

*OR* odds ratio, *aOR* adjusted odds ratio

^a^Missing data has been excluded

^b^Multi-level framework to adjust for across survey duplicates

**Table 8:** Univariate and multi-variate models assessing the impact of the COVID-19 pandemic on HIV testing rates among *people prescribed OAT* in all of Glasgow, 2010–2021 (June 2021 only)

Co-variates	N^a^	HIV test in the last year (% of N)	Univariate^b^		Multi-variate^b^	
			OR (95% CI)	P value	aOR (95% CI)	P value
Time period^a^						
Pre-outbreak (2010–2014)	11,908	1,973 (17%)	0.17 (0.16–0.18)	< 0.001	1.16 (0.15–0.17)	< 0.001
Early outbreak (2015–2016)	9,519	3,430 (36%)	0.48 (0.46–0.51)	< 0.001	0.47 (0.44–0.49)	< 0.001
Ongoing outbreak (2017–2019)	9,412	5,053 (54%)	1		1	
COVID-19 (2020–2021)	7,599	2,847 (37%)	0.52 (0.49–0.55)	< 0.001	0.50 (0.48–0.53)	< 0.001
Local authority area						
Rest of Glasgow	3,788	1,910 (50%)	1		1	
Glasgow city	10,410	6,198 (59%)	1.42 (1.34–1.50)	< 0.001	1.59 (1.49–1.68)	< 0.001
Gender						
Male	9,783	5,659 (58%)	1		1	
Female	4,415	2,449 (55%)	0.93 (0.89–0.98)	0.009	0.92 (0.87–0.97)	< 0.001
Age group^a^						
< 35	6,738	2,312 (34%)	1		1	
35–45	17,801	5,960 (35%)	1.03 (0.96–1.09)	0.437	0.80 (0.75–1.68)	< 0.001
46 +	14,619	5,031 (34%)	1.00 (0.94–1.07)	0.895	0.61 (0.56–0.65)	< 0.001
Injecting-related hospital admission in the last 2 years^a^						
No	29,457	9,046 (31%)	1		1	
Yes	8,981	4,257 (47%)	2.65 (0.51–2.80)	< 0.001	2.51 (2.37–2.66)	< 0.001

*OR* odds ratio, *aOR* adjusted odds ratio

^a^Time varying co-variate; people can be included in multiple categories

^b^Multi-level framework to adjust for across time period duplicates

**Table 9:** Univariate and multi-variate models assessing the impact of the COVID-19 pandemic on HIV testing rates among *people prescribed OAT* in Glasgow city, 2010–2021 (June 2021 only)

Co-variates	N^a^	HIV test in the last year (% of N)	Univariate^b^		Multi-variate^b^	
			OR (95% CI)	P value	aOR (95% CI)	P value
Time period^a^						
Pre-outbreak (2010–2014)	8,846	1,619 (18%)	0.17 (0.15–0.18)	< 0.001	0.16 (0.14–0.17)	< 0.001
Early outbreak (2015–2016)	6,956	2,690 (39%)	0.47 (0.43–0.49)	< 0.001	0.45 (0.42–0.48)	< 0.001
Ongoing outbreak (2017–2019)	6,837	3,930 (57%)	1		1	
COVID-19 (2020–2021)	5,465	2,066 (38%)	0.45 (0.42–0.48)	< 0.001	0.43 (0.40–0.46)	< 0.001
Gender						
Male	7,221	4,355 (60%)	1		1	
Female	3,189	1,843 (58%)	0.93 (0.88–0.99)	0.018	0.90 (0.85–0.97)	0.002
Age group^a^						
< 35	4,462	1,627 (38%)	1		1	
35–45	12,133	4,462 (37%)	0.94 (0.87–1.02)	0.130	0.76 (0.70–0.83)	< 0.001
46 +	11,709	4,216 (36%)	0.91 (0.84–0.99)	0.021	0.59 (0.54–0.64)	< 0.001
Injecting-related hospital admission in the last 2 years^a^						
No	21,233	6,883 (32%)	1		1	
Yes	6,881	3,422 (50%)	2.84 (2.67–3.03)	< 0.001	2.68 (2.50–2.87)	< 0.001

*OR* odds ratio, *aOR* adjusted odds ratio

^a^Time varying co-variate; people can be included in multiple categories

^b^Multi-level framework to adjust for across time period duplicates

**Table 10:** Univariate and multi-variate models assessing the impact of the COVID-19 pandemic on HIV testing rates among *people prescribed OAT* in rest of Glasgow, 2010–2021 (June 2021 only)

Co-variates	N^a^	HIV test in the last year (% of N)	Univariate^b^		Multi-variate^b^	
			OR (95% CI)	P value	aOR (95% CI)	P value
Time period^a^						
Pre-outbreak (2010–2014)	3,062	354 (12%)	0.17 (0.15–0.19)	< 0.001	0.16 (0.14–0.18)	< 0.001
Early outbreak (2015–2016)	2,563	740 (29%)	0.52 (0.47–0.58)	< 0.001	0.51 (0.46–0.57)	< 0.001
Ongoing outbreak (2017–2019)	2,575	1,123 (44%)	1		1	
COVID-19 (2020–2021)	2,134	781 (37%)	0.74 (0.67–0.83)	< 0.001	0.76 (0.68–0.85)	< 0.001
Gender						
Male	2,562	1,304 (51%)	1		1	
Female	1,226	606 (49%)	0.97 (0.87–1.07)	0.513	0.94 (0.85–1.05)	0.291
Age group^a^						
< 35	2,476	685 (28%)	1		1	
35–45	4,948	1,498 (30%)	1.14 (1.01–1.28)	0.036	0.88 (0.77–0.99)	0.035
46 +	2,910	815 (28%)	1.02 (0.89–1.16)	0.802	0.63 (0.55–0.74)	< 0.001
Injecting-related hospital admission in the last 2 years^a^						
No	8,234	2,163 (26%)	1		1	
Yes	2,100	835 (40%)	2.19 (1.97–2.45)	< 0.001	2.15 (1.92–2.42)	< 0.001

*OR* odds ratio, *aOR* adjusted odds ratio

^a^Time varying co-variate; people can be included in multiple categories

^b^Multi-level framework to adjust for across time period duplicates

**Table 11:** Univariate and multi-variate models assessing the impact of the COVID-19 pandemic on HIV testing rates among *people hospitalised for an injecting-related hospital admission (IRHA)* in all of Glasgow, 2010–2021 (June 2021 only)

Co-variates	N	HIV test in the last year (% of N)	Univariate^b^		Multi-variate^b^	
			OR (95% CI)	P value	aOR (95% CI)	P value
Time period^a^						
Pre-outbreak (2010–2014)	4,826	737 (15%)	0.24 (0.22–0.27)	< 0.001	0.19 (0.17–0.21)	< 0.001
Early outbreak (2015–2016)	3,347	1,082 (32%)	0.64 (0.59–0.71)	< 0.001	0.54 (0.49–0.60)	< 0.001
Ongoing outbreak (2017–2019)	5,297	2,248 (42%)	1		1	
COVID-19 (2020–2021)	3,008	1,411 (47%)	1.20 (1.10–1.30)	< 0.001	1.19 (1.08–1.31)	0.001
Council area						
Rest of Glasgow	4,438	1,161 (26%)	1		1	
Glasgow city	8,107	3,197 (39%)	1.91 (1.77–2.06)	< 0.001	1.83 (1.68–1.99)	< 0.001
Gender						
Male	8,885	3,128 (35%)	1		1	
Female	3,660	1,230 (34%)	0.93 (0.86–1.01)	0.068	0.91 (0.84–0.99)	0.032
Age group^a^						
< 35	5,964	1,332 (22%)	1		1	
35–45	5,537	2,281 (41%)	2.43 (2.23–2.65)	< 0.001	1.25 (1.14–1.38)	< 0.001
46 +	4,977	1,865 (37%)	2.08 (1.91–2.27)	< 0.001	0.94 (0.84–1.04)	0.241
Methadone prescribing^a^						
Not prescribed	7,373	1,194 (16%)	1		1	
In the last 6 months	7,620	3,631 (48%)	4.71 (4.34–5.10)	< 0.001	5.32 (4.83–5.85)	< 0.001
In the past but not the last 6 months	1,485	653 (44%)	4.06 (3.59–4.59)	< 0.001	4.34 (3.78–4.98)	< 0.001

*OR* odds ratio, *aOR* adjusted odds ratio

^a^Time varying co-variate; people can be included in multiple categories

^b^Multi-level framework to adjust for across time period duplicates

**Table 12:** Univariate and multi-variate models assessing the impact of the COVID-19 pandemic on HIV testing rates among *people hospitalised for an injecting-related hospital admission (IRHA)* in Glasgow city, 2010–2021 (June 2021 only)

Co-variates	N	HIV test in the last year (% of N)	Univariate^b^		Multi-variate^b^	
			OR (95% CI)	P value	aOR (95% CI)	P value
Time period^a^						
Pre-outbreak (2010–2014)	3,056	557 (18%)	0.24 (0.21–0.30)	< 0.001	0.18 (0.16–0.20)	< 0.001
Early outbreak (2015–2016)	2,149	807 (38%)	0.65 (0.58–0.72)	< 0.001	0.52 (0.46–0.58)	< 0.001
Ongoing outbreak (2017–2019)	3,474	1,671 (48%)	1		1	
COVID-19 (2020–2021)	2,082	1,055 (51%)	1.11 (1.00–1.23)	0.049	1.11 (0.98–1.24)	0.080
Gender						
Male	5,848	2,315 (39%)	1		1	
Female	2,259	882 (39%)	0.99 (0.90–1.09)	0.870	0.97 (0.87–1.07)	0.650
Age group^a^						
< 35	3,636	902 (25%)	1		1	
35–45	3,677	1,703 (46%)	2.61 (2.35–2.90)	< 0.001	1.37 (1.22–1.55)	< 0.001
46 +	3,448	1,485 (43%)	2.29 (2.06–2.55)	< 0.001	0.98 (0.87–1.11)	0.780
Methadone prescribing^a^						
Not prescribed	4,506	841 (19%)	1		1	
In the last 6 months	5,251	2,768 (53%)	4.86 (4.41–5.35)	< 0.001	5.63 (5.01–6.32)	< 0.001
In the past but not the last 6 months	1,004	481 (48%)	4.01 (3.45–4.65)	< 0.001	4.40 (3.72–5.21)	< 0.001

*OR* odds ratio, *aOR* adjusted odds ratio

^a^Time varying co-variate; people can be included in multiple categories

^b^Multi-level framework to adjust for across time period duplicates

**Table 13:** Univariate and multi-variate models assessing the impact of the COVID-19 pandemic on HIV testing rates among *people hospitalised for an injecting-related hospital admission (IRHA)* in rest of Glasgow, 2010–2021 (June 2021 only)

Co-variates	N	HIV test in the last year (% of N)	Univariate^b^		Multi-variate^b^	
			OR (95% CI)	P value	aOR (95% CI)	P value
Time period^a^						
Pre-outbreak (2010–2014)	1,770	180 (10%)	0.24 (0.20–0.29)	< 0.001	0.21 (0.17–0.25)	< 0.001
Early outbreak (2015–2016)	1,198	275 (23%)	0.64 (0.55–0.76)	< 0.001	0.57 (0.48–0.68)	< 0.001
Ongoing outbreak (2017–2019)	1,823	577 (32%)	1		1	
COVID-19 (2020–2021)	926	356 (38%)	1.35 (1.15–1.57)	< 0.001	1.40 (1.19–1.66)	< 0.001
Gender						
Male	3,037	813 (27%)	1		1	
Female	1,401	348 (25%)	0.87 (0.76–1.01)	0.061	0.80 (0.69–0.93)	0.004
Age group^a^						
< 35	2,328	430 (18%)	1		1	
35–45	1,860	578 (31%)	1.99 (0.71–2.31)	< 0.001	1.05 (0.88–1.24)	0.599
46 +	1,529	380 (25%)	1.45 (1.23–1.73)	< 0.001	0.85 (0.71–2.02)	0.087
Methadone prescribing^a^						
Not prescribed	2,867	353 (12%)	1		1	
In the last 6 months	2,369	863 (36%)	4.08 (3.53–2.47)	< 0.001	4.69 (3.97–5.55)	< 0.001
In the past but not the last 6 months	481	172 (36%)	3.96 (3.18–4.93)	< 0.001	4.16 (3.27–5.31)	< 0.001

*OR* odds ratio, *aOR* adjusted odds ratio

^a^Time varying co-variate; people can be included in multiple categories

^b^Multi-level framework to adjust for across time period duplicates

**Table 14:** Post-hoc analysis: multi-variate model including an interaction between time period and being hospitalised for an injecting-related admission in the last 2 years among *people prescribed OAT* in all of Glasgow, 2010–2021 (June 2021 only)

Co-variates	N^a^	HIV test in the last year (% of N)	Multi-variate^b^	
			aOR (95% CI)	P value
Injecting-related hospital admission in the last 2 years—NO				
Time period^a^				
Pre-outbreak (2010–2014)	10,354	1,532 (15%)	0.16 (0.15–0.17)	< 0.001
Early outbreak (2015–2016)	7,819	2,560 (33%)	0.48 (0.45–0.51)	< 0.001
Ongoing outbreak (2017–2019)	7,359	3,613 (49%)	1	
COVID-19 (2020–2021)	5,829	1,804 (31%)	0.47 (0.44–0.51)	< 0.001
Injecting-related hospital admission in the last 2 years—YES				
Pre-outbreak (2010–2014)	1,554	411 (28%)	2.37 (2.09–2.67)	< 0.001
Early outbreak (2015–2016)	1,700	870 (51%)	2.19 (1.97–2.44)	< 0.001
Ongoing outbreak (2017–2019)	2,053	1440 (70%)	2.44 (2.19–2.71)	< 0.001
COVID-19 (2020–2021)	1,770	1,043 (59%)	3.15 (2.81–3.51)	< 0.001
Local authority area				
Rest of Glasgow	3,788	1,910 (50%)	1	
Glasgow city	10,410	6,198 (59%)	1.58 (1.49–1.68)	< 0.001
Gender				
Male	9,783	5,659 (58%)	1	
Female	4,415	2,449 (55%)	0.91 (0.86–0.97)	0.002
Age group^a^				
< 35	6,738	2,312 (34%)	1	
35–45	17,801	5,960 (35%)	0.80 (0.75–0.86)	< 0.001
46 +	14,619	5,031 (34%)	0.61 (0.56–0.65)	0.002

*OR* odds ratio, *aOR* adjusted odds ratio

^a^Time varying co-variate; people can be included in multiple categories

^b^Multi-level framework to adjust for across time period duplicates

## References

[1] Degenhardt L, Webb P, Colledge-Frisby S (2023). Epidemiology of injecting drug use, prevalence of injecting-related harm, and exposure to behavioural and environmental risks among people who inject drugs: a systematic review. Lancet Glob Health.

[2] Larney S, Peacock A, Leung J (2017). Global, regional, and country-level coverage of interventions to prevent and manage HIV and hepatitis C among people who inject drugs: a systematic review. Lancet Glob Health.

[3] Wiessing L, Ferri M, Běláčková V (2017). Monitoring quality and coverage of harm reduction services for people who use drugs: a consensus study. Harm Reduct J BioMed Central.

[4] UK Health and Security Agency. Shooting Up: infections and other injecting-related harms among people who inject drugs in the UK, data to end of 2021 [Internet]. GOV.UK. 2023. https://www.gov.uk/government/publications/shooting-up-infections-among-people-who-inject-drugs-in-the-uk/shooting-up-infections-and-other-injecting-related-harms-among-people-who-inject-drugs-in-the-uk-data-to-end-of-2021. Accepted 4 Apr 2023

[5] UNAIDS. Understanding fast-track: accelerating action to end the AIDS epidemic by 2030 [Internet]. 2015. https://www.unaids.org/sites/default/files/media_asset/201506_JC2743_Understanding_FastTrack_en.pdf

[6] Ghys PD, Williams BG, Over M, Hallett TB, Godfrey-Faussett P (2018). Epidemiological metrics and benchmarks for a transition in the HIV epidemic. PLoS Med.

[7] Granich RM, Gilks CF, Dye C, De Cock KM, Williams BG (2009). Universal voluntary HIV testing with immediate antiretroviral therapy as a strategy for elimination of HIV transmission: a mathematical model. The Lancet.

[8] Brown LB, Spinelli MA, Gandhi M (2021). The interplay between HIV and COVID-19: summary of the data and responses to date. Curr Opin HIV AIDS.

[9] Stover J, Glaubius R, Teng Y (2021). Modeling the epidemiological impact of the UNAIDS 2025 targets to end AIDS as a public health threat by 2030. PLOS Med.

[10] McAuley A, Palmateer NE, Goldberg DJ, et al. Re-emergence of HIV related to injecting drug use despite a comprehensive harm reduction environment: a cross-sectional analysis. Lancet HIV [Internet]. Elsevier. 2019. http://www.ncbi.nlm.nih.gov/pubmed/30981674. Accessed 23 Apr 2019 10.1016/S2352-3018(19)30036-0 30981674

[11] Grimshaw C, Trayner KMA (2022). Editorial on ‘Epidemiology of HIV infection and associated behaviours among people who inject drugs in England, Wales, and Northern Ireland: nearly 40 years on’. HIV Med.

[12] Des Jarlais DC, Sypsa V, Feelemyer J (2020). HIV outbreaks among people who inject drugs in Europe, North America, and Israel. Lancet HIV.

[13] Gonsalves GS, Crawford FW (2018). Dynamics of the HIV outbreak and response in Scott County, IN, USA, 2011–15: a modelling study. Lancet HIV.

[14] Sypsa V, Psichogiou M, Paraskevis D (2017). Rapid decline in HIV incidence among persons who inject drugs during a fast-track combination prevention program after an HIV outbreak in Athens. J Infect Dis.

[15] Trayner KMA, Palmateer NE, McAuley A (2021). Evaluation of the scale-up of HIV testing among people who inject drugs in Scotland in the context of an ongoing HIV outbreak. Int J Drug Policy.

[16] Trayner, McAuley A, Palmateer NE, et al. Examining the impact of the first wave of COVID-19 and associated control measures on interventions to prevent blood-borne viruses among people who inject drugs in Scotland: an interrupted time series study|Elsevier Enhanced Reader. Drug Alcohol Depend [Internet]. 2022. https://reader.elsevier.com/reader/sd/pii/S0376871621007584?token=FF462509E58CB24654414A10B07B43560D70E913DB75009B7BF90E7A894CE8A1492898732C08F2CA7B7E08B91D942633&originRegion=eu-west-1&originCreation=20230303095944. Accessed 3 Mar 2023 10.1016/j.drugalcdep.2021.109263 PMC8802039 35120807

[17] Wiessing L, Sypsa V, Abagiu AO (2022). Impact of COVID-19 & response measures on HIV-HCV prevention services and social determinants in people who inject drugs in 13 sites with recent HIV outbreaks in Europe, North America and Israel. AIDS Behav.

[18] Public Health England. The impact of the COVID-19 pandemic on prevention, testing, diagnosis and care for sexually transmitted infections, HIV and viral hepatitis in England. 2020.

[19] Croxford S, Emanuel E, Shah A (2022). Epidemiology of HIV infection and associated behaviours among people who inject drugs in England, Wales, and Northern Ireland: nearly 40 years on. HIV Med.

[20] Chow EPF, Ong JJ, Denham I, Fairley CK (2021). HIV testing and diagnoses during the COVID-19 pandemic in Melbourne, Australia. J Acquir Immune Defic Syndr.

[21] Ejima K, Koizumi Y, Yamamoto N (2021). HIV testing by Public Health Centers and Municipalities and new HIV cases during the COVID-19 pandemic in Japan. J Acquir Immune Defic Syndr.

[22] Center for Disease Control. HIV Testing Before and During the COVID-19 Pandemic United States, 2019–2020. MMWR Morb Mortal Wkly Rep [Internet]. 2022. https://www.cdc.gov/mmwr/volumes/71/wr/mm7125a2.htm. Accessed 6 Apr 2023 10.15585/mmwr.mm7125a2 35737573

[23] Public Health Scotland. HIV in Scotland: update to 31 December 2021 [Internet]. 2022. https://publichealthscotland.scot/media/16641/hiv-infection-in-scotland-update-to-31-december-2021-main-report.pdf

[24] Public Health Agency of Canada. Tracks survey of people who inject drugs in Canada, Phase 4, 2017–2019: National findings, [Internet]. https://www.canada.ca/en/public-health/services/reports-publications/canada-communicable-disease-report-ccdr/monthly-issue/2020-46/issue-5-may-7-2020/survey-report-people-who-inject-drugs-canada-2017-2019.html. Accessed Jul 2020 10.14745/ccdr.v46i05a07 PMC8868043 35283692

[25] Public Health Scotland. The Needle Exchange Surveillance Initiative. 2022.

[26] The Kirby Institude. Australian NSP Survey National Data Report 2017–2021 [Internet]. 2022. https://kirby.unsw.edu.au/report/australian-nsp-survey-national-data-report-2017-2021. Accessed Jul 2022

[27] UK Health and Security Agency. Unlinked Anonymous Monitoring (UAM) Survey of HIV and viral hepatitis among PWID, 2022 report [Internet]. 2022. C:/Users/kirstt05/Zotero/storage/GE27E5SF/people-who-inject-drugs-hiv-and-viral-hepatitis-monitoring.html

[28] Alvarez-Madrazo S, McTaggart S, Nangle C, Nicholson E, Bennie M (2016). Data resource profile: the Scottish national prescribing information system (PIS). Int J Epidemiol.

[29] McAuley A, Fraser R, Glancy M, et al. Mortality among individuals prescribed opioid-agonist therapy in Scotland, UK, 2011–20: a national retrospective cohort study. Lancet Public Health [Internet]. 2023. https://www.thelancet.com/journals/lanpub/article/PIIS2468-2667(23)00082-8/fulltext. Accessed 19 Jul 2023 10.1016/S2468-2667(23)00082-8 37295452

[30] Larney S, Peacock A, Mathers BM, Hickman M, Degenhardt L (2017). A systematic review of injecting-related injury and disease among people who inject drugs. Drug Alcohol Depend.

[31] Gordon RJ, Lowy FD (2005). Bacterial infections in drug users. N Engl J Med.

[32] Information Services Division. Community Health Index (CHI) number [Internet]. 2021. https://www.ndc.scot.nhs.uk/Data-Dictionary/SMR-Datasets//Patient-Identification-and-Demographic-Information/Community-Health-Index-Number/. Accessed 6 May 2021

[33] Rogers W (1998). Regression standard errors in clustered samples. Stata Tech Bull.

[34] Williams RL (2000). A note on robust variance estimation for cluster-correlated data. Biometrics.

[35] Krawczyk N, Fawole A, Yang J, Tofighi B (2021). Early innovations in opioid use disorder treatment and harm reduction during the COVID-19 pandemic: a scoping review. Addict Sci Clin Pract.

[36] Trujols J, Larrabeiti A, Sànchez O, Madrid M, De Andrés S, Duran-Sindreu S (2020). Increased flexibility in methadone take-home scheduling during the COVID-19 pandemic: should this practice be incorporated into routine clinical care?. J Subst Abuse Treat.

[37] Brothers S, Viera A, Heimer R (2021). Changes in methadone program practices and fatal methadone overdose rates in connecticut during COVID-19. J Subst Abuse Treat.

[38] Figgatt MC, Salazar Z, Day E, Vincent L, Dasgupta N (2021). Take-home dosing experiences among persons receiving methadone maintenance treatment during COVID-19. J Subst Abuse Treat.

[39] Lintzeris N, Deacon RM, Hayes V (2022). Opioid agonist treatment and patient outcomes during the COVID-19 pandemic in south east Sydney, Australia. Drug Alcohol Rev.

[40] Croxford S, Emanuel E, Ibitoye A (2021). Preliminary indications of the burden of COVID-19 among people who inject drugs in England and Northern Ireland and the impact on access to health and harm reduction services. Public Health.

[41] Santos G-M, Ackerman B, Rao A (2021). Economic, mental health, HIV prevention and HIV treatment impacts of COVID-19 and the COVID-19 response on a global sample of cisgender gay men and other men who have sex with men. AIDS Behav.

[42] Miller RL, McLaughlin A, Montoya V, et al. Impact of SARS-CoV-2 lockdown on expansion of HIV transmission clusters among key populations: A retrospective phylogenetic analysis. Lancet Reg Health—Am [Internet]. 2022. https://www.thelancet.com/journals/lanam/article/PIIS2667-193X(22)00186-7/fulltext. Accessed 18 Jan 2023 10.1016/j.lana.2022.100369 PMC9500205 36168656

[43] Trayner KMA, McAuley A, Palmateer NE (2022). Examining the impact of the first wave of COVID-19 and associated control measures on interventions to prevent blood-borne viruses among people who inject drugs in Scotland: an interrupted time series study. Drug Alcohol Depend.

[44] Metcalfe R, Ragonnet-Cronin M, Bradley-Stewart A (2020). From hospital to the community: redesigning the Human Immunodeficiency Virus (HIV) clinical service model to respond to an outbreak of HIV among people who inject drugs. J Infect Dis.

[45] Herrmann ES, Matusiewicz AK, Stitzer ML, Higgins ST, Sigmon SC, Heil SH (2016). Contingency management interventions for HIV, tuberculosis, and hepatitis control among individuals with substance use disorders: a systematized review. J Subst Abuse Treat.

[46] Rumble C, Pevalin DJ, O’Moore É (2015). Routine testing for blood-borne viruses in prisons: a systematic review. Eur J Public Health.

[47] Bartholomew TS, Tookes HE, Serota DP, Behrends CN, Forrest DW, Feaster DJ (2020). Impact of routine opt-out HIV/HCV screening on testing uptake at a syringe services program: an interrupted time series analysis. Int J Drug Policy.

[48] Drug Deaths Taskforce. Medication Assisted Treatment (MAT) Standards for Scotland. 2020.

[49] NHS England. Emergency department opt out testing for HIV, hepatitis B and hepatitis C: the first 100 days [Internet]. 2022. https://www.england.nhs.uk/long-read/emergency-department-opt-out-testing-for-hiv-hepatitis-b-and-hepatitis-c-the-first-100-days/

[50] Scottish Government. Ending HIV transmission in Scotland by 2030 [Internet]. 2022. http://www.gov.scot/publications/ending-hiv-transmission-scotland-2030/. Accessed 6 Apr 2023

